# Development of dual inhibitors targeting DprE1 and AHAS for treatment of *Mycobacterium tuberculosis* infection

**DOI:** 10.1186/1471-2334-14-S3-E24

**Published:** 2014-05-27

**Authors:** Rupesh V Chikhale, Amit M Pant, Sunil S Menghani, Pramod B Khedekar

**Affiliations:** 1 Department of Pharmaceutical Sciences, Rashtrasant Tukadoji Maharaj Nagpur University, Nagpur, India

## Background

The emerging multidrug resistant (MDR) and extensively drug resistant (XDR) *Mycobacterium tuberculosis* (MTB) infection is increasing with greater complexity, estimated 220000-400000 tuberculosis cases emerged in 2011 globally. A number of lead compounds have been developed for treatment of MDR and XDR TB, but no new chemical entity has emerged for clinical use. Recently DprE1 and AHAS have been identified as promising drug targets.

## Methods

The methodology involved *in silico* studies, synthesis and *in vitro* evaluation for inhibition of *M. tuberculosis*. *In silico* studies involved protein preparation for DprE1, AHAS, docking and analysis of docking results. Sixty two substituted (thiazolidine-2-yl amino) benzthiazolylphenylhydrazine carbothiamide derivatives were studied. *In vitro* evaluation was carried out by modified agar diffusion method.

## Results

About 62 compounds were synthesized based on molecular docking studies. In case of DprE1 maximum interactions were found with His132, Asn385, Gly133, Leu134, Leu363, Val365, whereas in case of AHAS maximum interactions were shown between Arg318, Gly138, Lys197, Trp516 and Phe147. All compounds were synthesized in satisfactory yield and structurally elucidated. The range of MIC was found between 40-80 mg/L with percentage inhibition in range of 80-95%.

## Conclusion

Experimental results reveales that newly developed compounds exhibited promising antitubercular activity which can be further explored for development of potent drugs.

